# Impact of a Digital Scribe System on Clinical Documentation Time and Quality: Usability Study

**DOI:** 10.2196/60020

**Published:** 2024-09-23

**Authors:** Marieke Meija van Buchem, Ilse M J Kant, Liza King, Jacqueline Kazmaier, Ewout W Steyerberg, Martijn P Bauer

**Affiliations:** 1 CAIRELab (Clinical AI Implementation and Research Lab) Leiden University Medical Center Leiden Netherlands; 2 Department of Digital Health University Medical Center Utrecht Utrecht Netherlands; 3 Autoscriber B.V. Eindhoven Netherlands; 4 Department of Biomedical Data Sciences Leiden University Medical Center Leiden Netherlands; 5 Department of Internal Medicine Leiden University Medical Center Leiden Netherlands

**Keywords:** large language model, large language models, LLM, LLMs, natural language processing, NLP, deep learning, pilot study, pilot studies, implementation, machine learning, ML, artificial intelligence, AI, algorithm, algorithms, model, models, analytics, practical model, practical models, automation, automate, documentation, documentation time, documentation quality, clinical documentation

## Abstract

**Background:**

Physicians spend approximately half of their time on administrative tasks, which is one of the leading causes of physician burnout and decreased work satisfaction. The implementation of natural language processing–assisted clinical documentation tools may provide a solution.

**Objective:**

This study investigates the impact of a commercially available Dutch digital scribe system on clinical documentation efficiency and quality.

**Methods:**

Medical students with experience in clinical practice and documentation (n=22) created a total of 430 summaries of mock consultations and recorded the time they spent on this task. The consultations were summarized using 3 methods: manual summaries, fully automated summaries, and automated summaries with manual editing. We then randomly reassigned the summaries and evaluated their quality using a modified version of the Physician Documentation Quality Instrument (PDQI-9). We compared the differences between the 3 methods in descriptive statistics, quantitative text metrics (word count and lexical diversity), the PDQI-9, Recall-Oriented Understudy for Gisting Evaluation scores, and BERTScore.

**Results:**

The median time for manual summarization was 202 seconds against 186 seconds for editing an automatic summary. Without editing, the automatic summaries attained a poorer PDQI-9 score than manual summaries (median PDQI-9 score 25 vs 31, *P*<.001, ANOVA test). Automatic summaries were found to have higher word counts but lower lexical diversity than manual summaries (*P*<.001, independent *t* test). The study revealed variable impacts on PDQI-9 scores and summarization time across individuals. Generally, students viewed the digital scribe system as a potentially useful tool, noting its ease of use and time-saving potential, though some criticized the summaries for their greater length and rigid structure.

**Conclusions:**

This study highlights the potential of digital scribes in improving clinical documentation processes by offering a first summary draft for physicians to edit, thereby reducing documentation time without compromising the quality of patient records. Furthermore, digital scribes may be more beneficial to some physicians than to others and could play a role in improving the reusability of clinical documentation. Future studies should focus on the impact and quality of such a system when used by physicians in clinical practice.

## Introduction

In recent years, the issue of burnout among physicians has been increasingly recognized within the health care sector. A survey conducted in 2017 involving 5000 physicians in the United States found that 44% exhibited at least 1 sign of burnout [1]. In response to this issue, the National Academy of Medicine established a committee dedicated to enhancing patient care through the promotion of physician well-being. The committee produced a detailed report titled Taking Action Against Clinician Burnout, which outlines the causes of burnout among physicians. A significant cause identified is the growing administrative workload [2]. The introduction of the electronic health record (EHR) has led to physicians spending up to half of their working hours on administrative duties [3-5]. Such tasks have been shown to lower job satisfaction for physicians [6] and negatively impact the physician-patient relationship [7]. Additionally, research linking the use of EHR to burnout indicates that physicians spending more time on EHR, particularly outside of regular hours, face a greater risk of experiencing burnout [8,9].

Recent advances in natural language processing (NLP) have created the possibility of automating some of these administrative tasks. One of these promises is the creation of the so-called “digital scribe.” Such a system, first described in 2018, automatically records, transcribes, and summarizes the clinical encounter [10,11]. A scoping review from 2022 presented an overview of the capabilities of digital scribes at that point in time, and showed that none of these systems had the full capability of a digital scribe [12]. The introduction of large language models has disrupted this field, with many papers describing their potential value in clinical note generation and multiple companies now offering digital scribe systems [13-15]. However, an evaluation on the potential impact of such a system on documentation time, including the assessment of quality and user experiences is not available to date. A thorough, prospective investigation of digital scribe performance and impact on routine practice is necessary to ensure the safety and effectiveness of the system. The aim of the current study is to assess the potential impact on the time spent and quality of medical summaries using a Dutch, commercially available digital scribe system.

## Methods

### Data

Our data set consisted of 27 recordings of mock consultations between physicians and nonmedical individuals. The consultations were structured around 26 vignettes, created by an internist. These vignettes delineated a set of symptoms, with a focus on various presentations of chest pain. Nonmedical individuals, assuming the role of patients, were provided with these vignettes. They were encouraged to develop and present a narrative surrounding the described symptoms. The participating physicians, all specialists in internal medicine from the Leiden University Medical Center, engaged with these simulated patients, applying their expertise to the scenarios presented. The average duration of the consultations was 293 (IQR 189-398) seconds.

### Participants

In total, 21 medical students with experience in clinical practice and clinical documentation from Leiden University Medical Center consented to participate in our study. All students had a bachelor’s degree in medicine and completed a course in clinical documentation. The students received a compensation of €100 (US $111) for their participation.

### Autoscriber

Autoscriber is a web-based software application that transcribes and summarizes medical conversations (currently with support for Dutch, English, and German). The pipeline uses a transformer-based speech-to-text model, fine-tuned on proprietary clinical data for transcription and a mixture of large language models such as GPT-3.5 and GPT-4, combined with a tailored prompt structure and additional rules for summarization. The tool also has self-learning functionality, which was not evaluated in this study for practical reasons.

### Summarization

**Figure 1:**
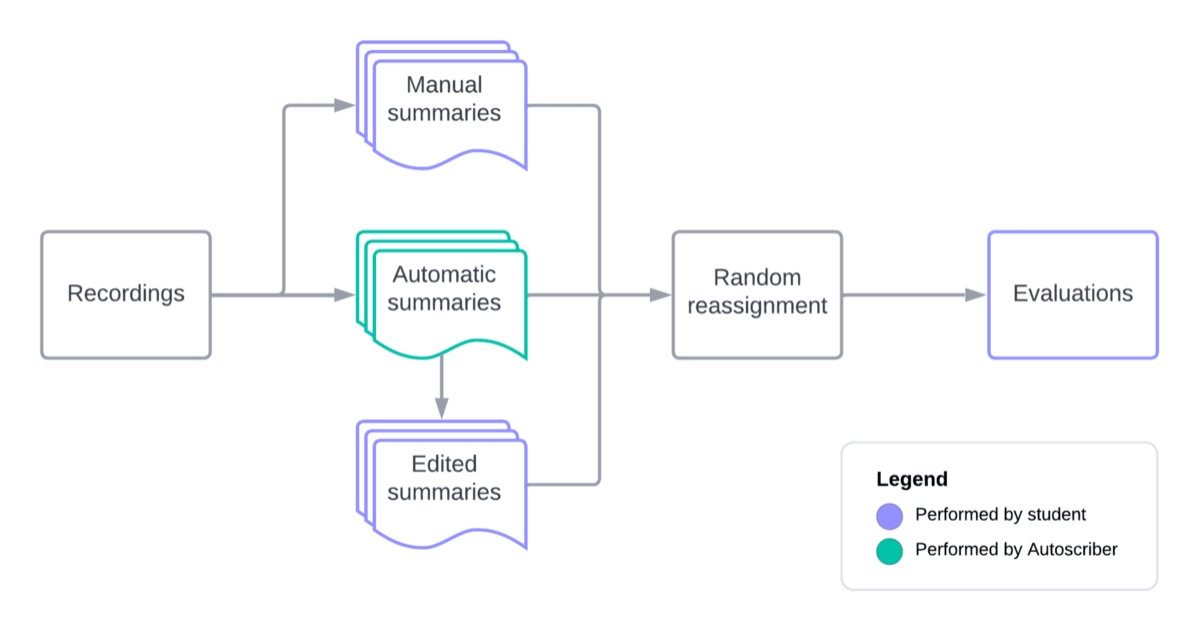
Flowchart showing the 3 different summarization methods and consecutive evaluation.

All students summarized 4 consultations manually, then 8 consultations using Autoscriber, and finally 4 consultations manually to minimize a learning effect (see Figure 1). In total, students summarized 16 unique consultations.

#### Manual Summarization

Students were asked to listen to the full recording, making some notes using pen and paper. At the end of the recording, they started timing and summarized the consultation on the computer. When finished, they recorded the total time spent summarizing.

#### Automatic Summarization

For the 8 consultations summarized using Autoscriber, the setup was similar. However, students first opened the Autoscriber application and, while listening to the recording, also recorded the consultation with Autoscriber. Once Autoscriber had created an automatic summary, students started timing and edited the automatic summary. Finally, they uploaded both the automatic summary and the edited summary, including the total time they spent editing.

### Evaluation

Once all summaries were created, the manual, automatic, and edited summaries were randomly reassigned to other students, who were blinded for the method used to create the summary. Students first listened to the full recording, and then evaluated the related summaries using a modified version of the Physician Documentation Quality Instrument (PDQI-9) [16]. The PDQI-9 is a validated evaluation instrument for assessing the quality of clinical documentation, consisting of 9 questions. We removed question 1 (up-to-date: the note contains the most recent test results and recommendations) and 8 (synthesized: the note reflects the author’s understanding of the patient’s status and ability to develop a plan of care) for our study, as these could not be answered in the current setup. We translated the questions into Dutch, which were reviewed by one clinician (MB). Per recording, we selected the manual summary with the highest PDQI-9 score as the reference standard summary.

At the end of the study, we asked students about their experience with Autoscriber, what was positive, what should be improved, and if they would want to use Autoscriber in their work. For a more in-depth view of the differences between the automatic and edited summaries, we prompted ChatGPT (paid version, GPT-4) to assess the differences. The prompt was created iteratively using PromptPerfect until the format of the answer was satisfactory. We then ran the prompt several times to check for internal consistency. Two researchers (MB and MvB) manually checked the answers provided by ChatGPT.

### Data Analysis

#### Preprocessing

For every summary, we calculated the total word count and the lexical diversity. Furthermore, to compare the automatic summaries to their edited counterparts we calculated the number of insertions, deletions, the Recall-Oriented Understudy for Gisting Evaluation (ROUGE)–1 and ROUGE-L score [17], and the BERTScore metric [18]. The ROUGE-1 score calculates the overlap in words between 2 texts. The ROUGE-L score calculates the longest common subsequence. The BERTScore metric uses contextual embeddings to compare words between 2 texts.

#### Power Analysis

To ensure the study was adequately powered to detect a large effect size (Cohen *f*=0.4) between 3 groups with an alpha level of 0.05 and a power of 95%, a power analysis was conducted using the FTestAnovaPower function from the statsmodels library in Python. This analysis assumed equal group sizes and did not account for potential correlations among repeated measures.

#### Statistical Analysis

The differences between the automatic and associated edited summaries were tested using a paired *t* test. To compare the differences in summaries per recording, we selected the manual summary with the highest PDQI-9 score as the reference standard. We then calculated the ROUGE-1 and ROUGE-L scores for all the other manual, automatic, and edited summaries. The differences in word count, lexical diversity, PDQI-9 score, and ROUGE scores between the 3 methods was tested using one-way ANOVA and, if the *P*-value was below .05, followed by Tukey Honestly Significant Difference test. To assess the possibility of a learning effect, we compared the first and second batch of manual summaries on time spent creating the summary and PDQI-9 score using a paired sample *t* test. We used Python for the analysis, using the “statsmodels” and the “scipy” package.

### Ethical Considerations

This study was conducted in accordance with the Declaration of Helsinki. For the purposes of this study, ethics approval was not applicable as the research did not include actual patients or any personal or sensitive information. All students involved in the study were informed about the purpose of the research, the use of the data, and gave their informed consent to participate in the study under these conditions.

## Results

The power analysis indicated that a sample size of approximately 100 participants per group would be necessary to achieve the desired power of 95% for detecting a large effect size among the 3 groups under the specified conditions. In total, we collected 156 manual summaries, 137 automatic summaries, and 137 edited summaries from 21 students. A difference in the total number of manual, automatic, and edited summaries occurred because 3 students dropped out of the study due to time restraints. Table 1 shows an example of a manual, automatic, and edited summary of the same recording. 18 students completed the evaluation phase of the study. The median time students spent creating or editing the summaries was 186 seconds (IQR 109-267). Summaries had a median length of 129 (IQR 91-172) words. On average, summaries had a median PDQI-9 score of 28.5 (IQR 25-32) out of a maximum of 35 (Table 2). Multimedia Appendix 1 shows an extended version of Table 2, including the results of the Tukey Honestly Significant Difference test. There was a difference in time spent on manually summarizing the first batch and the second batch, with a median of 246 (IQR 137-311) and 188 (IQR 118-226), respectively, (*P*=.004). However, there was no difference in PDQI-9 score between these 2 batches.

**Table 1:** An example of a manual, automatic, and edited summary of the same recording.

Manual summary (translated)	Automatic summary	Edited summary	
For a few days now, tearing pain in the chest with radiation to behind the shoulder blades. Was sitting on the couch watching TV, pain started acutely, felt like a tear and is continuously present. No complaints of tingling, numbness, or cold hands. No loss of function in arm or leg. No palpitations. Not nauseous. Has not had similar complaints before. The patient is on the “edge” of hypercholesterolemia, does not use medication. Measures blood pressure regularly for donation, pressure regular, last 120/75. The patient has never smoked. Drinks alcohol on the weekend. Family: Father had congenital vascular problems, began having heart problems at the beginning of his 50s, also had heart valve problems, died of heart failure. Brother had a heart attack at age 46, was stented. Both brothers have hypercholesterolemia. No connective tissue disorders in the family. The patient is worried due to familial CVD (Cardiovascular Disease).	Reason for referral: Main complaint: Persistent chest pain, feels as if something is tearing and radiates to behind the shoulder blades. History: Chest pain started a few days ago while calmly watching TV. The pain remains constantly present since the beginning. The pain feels as if something is tearing and radiates to the back of the shoulder blades. There are heart problems in the family (patient's father and brother). The patient's father had a poor vascular system and a leaking heart valve, and died of a heart failure. The patient's brother had a heart attack at the age of 46 and was stented. Social history: The patient does not smoke and drinks moderately alcohol on the weekend. Current medication: The patient does not use any medication. Physical examination: Additional investigation: Blood pressure is regularly measured and is about 75 over 120. The patient gives blood every six months. Diagnosis: Treatment plan: Requested examinations: Informed consent:	For several days now, tearing pain in the chest, radiating to between the shoulder blades. Chest pain started a few days ago while calmly watching TV and has been stable since then. No palpitations, not nauseous, no tingling or numb feeling. Blood pressure is regularly measured and is about 75 over 120. Cholesterol is good. Patient is worried because of family history. Fam: father had heart problems, brother had a heart attack at a young age, hypercholesterolemia, no connective tissue diseases. Intox: no smoking, alcohol on weekends in moderation Med: none	

**Table 2:** Descriptive statistics of the different methods and associated *P* values.

Metrics		Manual (n=156), median (IQR)	AS edited (n=137), median (IQR)	AS (n=137), median (IQR)	*P* value (ANOVA)
Time spent on summary (seconds)		202 (128-286)	152 (93-244)	0 (0-0)	<.001
Word count		101 (67-141)	137 (96-194)	148 (116-180)	<.001
Lexical diversity		0.68 (0.63-0.74)	0.61 (0.56-0.66)	0.59 (0.53-0.63)	<.001
**PDQI-9^a^ score**					
	Overall	31 (27-33)	29 (26-33)	25 (22-28)	<.001
	Accurate	5 (4-5)	5 (4-5)	4 (2-5)	<.001
	Thorough	4 (4-5)	4 (4-5)	3 (2-4)	<.001
	Useful	5 (4-5)	4 (4-5)	4 (3-4)	<.001
	Organized	4 (3-5)	4 (3-5)	4 (3-4)	.01
	Comprehensible	5 (4-5)	5 (4-5)	4 (3-5)	<.001
	Succinct	5 (4-5)	4 (2-5)	3 (2-4)	<.001
	Internally consistent	5 (4-5)	5 (4-5)	5 (4-5)	<.001
ROUGE^b,c^-1 *F*_1_-score		47.3 (42.5-56.4)	40.6 (35.0-45.4)	32.3 (27.0-37.4)	<.001
ROUGE-L *F*_1_-score		29.4 (23.7-37.6)	23.4 (20.6-27.5)	19.6 (15.7-23.5)	<.001
BERTScore^c^ *F*_1_-score		74.6 (71.9-77.0)	71.6 (69.5-73.7)	68.6 (67.5-70.3)	<.001

^a^PDQI-9: Physician Documentation Quality Instrument.

^b^ROUGE: Recall-Oriented Understudy for Gisting Evaluation.

^c^To calculate the ROUGE score and BERTScore, the highest scoring manual summary was taken as the reference standard. These summaries were taken out of the data set when calculating the average ROUGE scores.

### Comparison Between Automatic and Corresponding Edited Summaries

Students inserted a median of 45 (IQR 27-82) words and deleted 46 (IQR 27-80) words. The edits led to a median increase in PDQI-9 score of 4.0 (IQR 1-8). The median ROUGE-1 F1 score between the automatic and their corresponding edited summaries was 73.3 (IQR 61.0-84.4), the ROUGE-L F1 score was 67.4 (IQR 50.0-80.5), and the BERTScore F1 was 84.1 (IQR 79.0-89.4).

ChatGPT assessed the differences between automatic summaries and their edited counterparts on the following aspects: language use and precision, clarity and detail, coherence and flow, structural differences, stylistic variations, and the most common deletions and insertions. The final prompt can be seen in Multimedia Appendix 2. See Table 3 for the observations per aspect. The assessment by ChatGPT aligned with the sample analysis performed by the researchers. Furthermore, similar aspects were mentioned by the students.

**Table 3:** Differences between automatic and edited summaries, as assessed by ChatGPT.

Aspect	Automatic summaries	Edited summaries	Observations
Language use and precision	Generally simplistic and formulaic language. For example, “Chest pain started a few days ago while quietly watching TV.”	More sophisticated and precise language. Example: ”Since a few days tearing chest pain radiating to between the shoulder blades.“	Human editors refine the language to be more precise and contextually appropriate.
Clarity and detail	Often vague, lacking specific details. For instance, “Patient has had persistent watery diarrhea since one week.”	Provide clearer, more detailed descriptions. Example: “Patient has had persistent watery diarrhea for a week with a frequency of ten times a day.”	Human editing enhances clarity by adding relevant details that were omitted in the automatic summaries.
Coherence and flow	Sometimes disjointed or lacking in logical flow. Example: “The chest pain started suddenly and has been continuously present since it started.”	Better structured, with a smoother flow of ideas. Example: “The patient complains of sudden and persistent chest pain that started several days ago.”	Human editors improve the coherence, making the summaries easier to follow.
Structural differences	Tend to follow a predictable structure, possibly template-based.	More varied structures, adapted to the content's needs.	Human editing allows for more flexible structuring, tailored to the specific summary.
Stylistic variations	Limited stylistic variations, often repetitive.	Display a wider range of styles, adapting to the tone and context.	Human editors introduce stylistic diversity, making each summary more unique.
Most common deletions			Redundant phrases, overly general statements.
Most common insertions			Specific details, clarifying phrases, and contextual information.

### Differences Per Student

Using Autoscriber had a different effect per student. For 8 out of 18 students, using Autoscriber was associated with a decrease in PDQI-9 score, while for the other students the difference in PDQI-9 score between manual and automatic summaries had a *P* value above .05. For 5 students, editing the automatic summary took more time than manually creating a summary, although these differences were not significant. For 3 students, editing the automatic summary led to a decrease in time spent on summarizing, with a *P* value lower than .05. See Multimedia Appendix 3 for the full overview.

### Experiences With the Use of Autoscriber

Students were generally very positive about using Autoscriber, mentioning that it was nice or interesting to use (n=9), easy and simple in use (n=6), and that they believed in the potential of such a tool (n=4). Four students mentioned the automatic summary exceeded their expectations, while 4 other students said the quality of the summary was insufficient due to errors and the amount of time needed to make edits. A specific error that was mentioned multiple times was that the summary did not include negative symptoms (eg, the absence of shortness of breath). Three students mentioned the tool did not always work: it would sometimes load for a very long time or get stuck while generating the summary. This was due to limitations in graphics processing unit capacity at that time. See Table 4 for the positive aspects and points of improvement mentioned by the students. A majority of students (12/18, 67%) would want to use the application during their work. The other students (6/18, 33%) said they would want to use the application if improvements were made.

**Table 4:** Themes most often described by students about the positive aspects and points of improvement.

Mentioned aspects		Count
**Positive**		
	Easy to use	5
	Good accuracy, eg, amount of details, good use of language, low amount of errors, inclusion of important symptoms	5
	Summary fairly complete	4
	Saves time	4
	Well-structured view	4
	Nice to have something to start with, without typing	3
**Negative**		
	Structure does not align with preferences, eg, headings unclear, illogical structure, does not align with style	6
	Wordy/lengthy	5
	Relevant information missing, eg, details, absence of symptoms	5
	Comments on language use, eg, use of nonstandard words, vague descriptions, too literal, absence of common abbreviations	5
	Duration of summarization time	3
	Presence of irrelevant information	2

## Discussion

### Principal Findings

In this impact study, we extensively evaluated the efficacy of Autoscriber, a Dutch digital scribe system, in enhancing the clinical documentation process in a pilot setting. A group of trained medical students summarized clinical conversations with and without the tool. We found differences between automatic and manual summaries in time spent on the summary, the word count, lexical diversity, and qualitative aspects such as accurateness and usefulness. These differences decreased after students edited the automatic summaries. During editing, medical students most often added context and details, while removing overly general statements and irrelevant text. Most were positive about using the tool, although some mentioned the summaries were lengthy and the structure did not always align with their preferences.

As the first impact study of a fully functioning digital scribe system, we provide some interesting insights into the possible future of digital scribes in health care. First of all, we show that a collaboration between the system and the students leads to the best results at this point in time, with a decrease in time spent on summarizing in combination with a similar quality when compared to manual summarization. We believe the current setting might even provide an overestimation of the quality of the manual summaries: the students did not have a time cap for creating the summaries, while in clinical practice, physicians often have to create a summary during or in between consultations. Furthermore, multiple studies show a negative association between seniority of a physician and the completeness of a medical record [19-21]. Taking this into account, we see the potential in using a digital scribe system that provides a first draft, which the physician then edits. In the current setup, this collaboration led to a decrease in time spent summarizing, while keeping the quality of the summary on par.

When looking at the differences between the 3 methods, the higher word count and lower lexical diversity in the automatic summaries compared to the manual summaries stood out. Two previous studies compared human and ChatGPT-written medical texts and reported similar results [22,23]. Furthermore, one of these studies reported human texts contained more specific content, which we found as well. These aspects are essential to improve in future versions, as they directly link to the quality of a summary in terms of succinctness and thoroughness. An increased summary length could lead to an increase in time spent reading or analyzing summaries downstream in the clinical process. However, a small decrease in lexical diversity in combination with a more structured summary could also be seen as a step toward standardization of medical summaries. This aspect is becoming more important since clinical documentation is increasingly reused for other purposes, such as research and quality measurements. Furthermore, previous studies show that structured documentation leads to increased note quality [24], which in turn has been shown to positively affect the quality of care [25-27]. These potential effects have to be studied in future research.

We found large differences in the effect of using Autoscriber on PDQI-9 score and time spent summarizing between students. While using Autoscriber decreased the time spent on finalizing the summary for most students, there were a few students who spent more time on editing the automatic summary then on manually creating a summary. Furthermore, the difference in PDQI-9 score between manual and automatic summaries differed greatly between students. This result is highly relevant, as it shows that the added value of using a digital scribe differs per user. Future studies should investigate which users could gain most benefit in using a digital scribe, taking into account age, specialty, the ability to type blindly, and other factors that might impact the added value on a personal level.

### Strengths and Limitations

This impact study on a digital scribe system for clinical conversations presents a novel exploration into the practical application of such technology. Since the introduction of ChatGPT, many papers have described the potential of using ChatGPT and other large language models in health care. While their potential is clear, these models have still to prove their actual clinical value. This study takes a first step in gaining a better view of the potential effects such a digital scribe system could have on the documentation process, especially in interaction with the user. Apart from quantitative analyses, we also included several different qualitative analyses, providing a more in-depth view of the differences between the summaries and the experiences of the students. These results are highly relevant for researchers and companies developing digital scribes as well as health care organizations considering using a digital scribe in the near future.

One limitation is the setup of our study, which is not fully representative of clinical practice. Specifically, our reliance on medical students listening to prerecorded mock consultations does not fully capture the dynamic and often unpredictable nature of real-time clinical interactions. The controlled environment of our study does not account for the varied technological, environmental, and personal factors that can influence the use and effectiveness of digital scribe systems in live clinical environments. However, this approach allowed us to isolate and evaluate the impact on summarization time and differences in summary between the 3 methods. Future research should aim to incorporate real clinical interactions to validate and extend our findings.

Another limitation is the lack of a reference summary per consultation. To calculate the ROUGE scores, we designated the highest scoring manual summary as the reference standard per consultation. This method suffices for the current pilot study; however, it brings up the bigger issue of summary evaluation metrics. The ROUGE score remains the most used metric, while this metric only measures exact overlap in words and is, thus, very sensitive to the choice of reference summaries [28]. Because of this limitation, we added the BERTScore metric, which has been shown to correlate better with human evaluations [18]. However, the overall lack of a standard for clinical documentation still poses a considerable challenge for the objective assessment of summarization efficacy of digital scribes. This underscores the necessity for developing more sophisticated evaluation methods, especially with the arrival of large language models in health care.

### Future Implications

Our findings underscore the promising potential of integrating digital scribe technologies like Autoscriber within clinical settings to alleviate the administrative burdens faced by health care professionals. Future clinical impact studies are imperative to explore the broader effects of digital scribes on the physician-patient interaction, documentation accuracy, and overall health care delivery efficiency. These studies should aim to evaluate the real-world applicability of digital scribes, including their impact on clinical workflow, quality of care, and patient satisfaction. Especially the latter, which has not received sufficient attention up to now, should be the focus of future research to ensure the physician-patient relationship is not harmed. Additionally, exploring the customization of digital scribe systems to fit the specific needs and preferences of individual physicians or specialties could enhance user adoption and effectiveness. As the field of large language models is developing at a fast rate and digital scribes will improve quickly, repeated or continuous evaluation of these systems is necessary. A recent study described the development and evaluation of a chat-based diagnostic conversational agent [29]. This agent outperformed primary health care providers in both diagnosis and the development of a treatment plan. The introduction of digital scribes in clinical practice could eventually lead to similar support during the clinical encounter, where the digital scribe might suggest additional follow-up questions or provide a differential diagnosis. Ultimately, the goal is to seamlessly integrate digital scribes into clinical practice, ensuring they enhance patient care and physician well-being.

### Conclusions

This study explores the impact of a Dutch digital scribe system on the clinical documentation process, offering significant insights into its potential to enhance physicians’ experience. By demonstrating the use of the system in reducing summarization time while maintaining summary quality through collaborative editing, our research highlights the potential of digital scribe systems in addressing the challenges of clinical documentation. Despite the limitations related to the representativeness of our pilot setup and the evaluation of summary quality, the positive outcomes suggest a promising avenue for future research and development. Further studies, particularly those involving real-world clinical settings, are essential to fully understand the implications of digital scribes on the physician-patient dynamic and health care delivery.

## Appendix

Multimedia Appendix 1 Extended Table 2: Descriptive statistics of the different methods and associated *P* values.

Multimedia Appendix 2 Prompt provided to ChatGPT, version 4.0.

Multimedia Appendix 3 Differences in PDQI-9 (Physician Documentation Quality Instrument) score between automatic and manual summaries per student and in time spent on manual and edited summaries per student.

## Abbreviations

EHR: electronic health record
NLP: natural language processing
PDQI-9: Physician Documentation Quality Instrument
ROUGE: Recall-Oriented Understudy for Gisting Evaluation

## Data Availability

The majority of the data supporting the findings of this study are included in the manuscript. Additional data sets generated during and analyzed during the current study are not publicly available but are available from the corresponding author on reasonable request and subject to approval by the institutional review board.
