# Supplementary material for: Impact of a Digital Scribe System on Clinical Documentation Time and Quality: Usability Study
Source: JMIR AI. 2024 Sep 23;3:e60020. doi: 10.2196/60020 (PMC11459111; doi:10.2196/60020)
Supplement: Multimedia Appendix 1 [file ai_v3i1e60020_app1.docx]

**Extended Table 2: Descriptive statistics of the different methods and associated p-values.**

| Metrics  Median [IQR] | Manual  [n = 156] | AS edited  [n = 137] | AS  [n = 137] | P-value manual vs. AS | P-value manual vs. AS edited | P-value AS edited vs. AS |
| --- | --- | --- | --- | --- | --- | --- |
| Time spent on summary in seconds | 202 [128-286] | 152 [93-244] | 0 [0-0] | <0.001 | 0.02 | <0.001 |
| Word count | 101 [67-141] | 137 [96-194] | 148 [116-180] | 0.05 | <0.001 | 0.83 |
| Lexical diversity | 0.68 [0.63-0.74] | 0.61 [0.56-0.66] | 0.59 [0.53-0.63] | <0.001 | <0.001 | 0.01 |
| PDQI score  - Overall  - Accurate  - Thorough  - Useful  - Organized  - Comprehensible  - Succinct  - Internally consistent | 31 [27-33]  5 [4-5]  4 [4-5]  5 [4-5]  4 [3-5]  5 [4-5]  5 [4-5]  5 [4-5] | 29 [26-33]  5 [4-5]  4 [4-5]  4 [4-5]  4 [3-5]  5 [4-5]  4 [2-5]  5 [4-5] | 25 [22-28]  4 [2-5]  3 [2-4]  4 [3-4]  4 [3-4]  4 [3-5]  3 [2-4]  5 [4-5] | <0.001  <0.001  <0.001  <0.001  0.03  <0.001  <0.001  <0.001 | 0.16  0.99  0.96  0.10  0.89  0.34  <0.001  0.89 | <0.001  <0.001  <0.001  <0.001  0.01  <0.001  <0.001  <0.001 |
| ROUGE-1 (F1) | 47.3 [42.5-56.4] | 40.6 [35.0-45.4] | 32.3 [27.0-37.4] | <0.001 | <0.001 | <0.001 |
| ROUGE-L (F1) | 29.4 [23.7-37.6] | 23.4 [20.6-27.5] | 19.6 [15.7-23.5] | <0.001 | <0.001 | 0.06 |
| BERTScore (F1) | 74.6 [71.9-77.0] | 71.6 [69.5-73.7] | 68.6 [67.5-70.3] | <0.001 | <0.001 | <0.001 |
| Significance tested using Tukey’s HSD test.  To calculate the ROUGE, the highest scoring manual summary was taken as the reference standard. These summaries were taken out of the dataset when calculating the average ROUGE scores. | | | | | | |
