# Supplementary material for: Impact of a Digital Scribe System on Clinical Documentation Time and Quality: Usability Study
Source: JMIR AI. 2024 Sep 23;3:e60020. doi: 10.2196/60020 (PMC11459111; doi:10.2196/60020)
Supplement: Multimedia Appendix 2 [file ai_v3i1e60020_app2.docx]

## Prompt provided to ChatGPT, version 4.0

Perform a comparative analysis of two sets of summaries. The first set consists of automatically generated summaries, and the second set comprises the same summaries but edited by humans. Both sets can be found in the attached .docx document, where the automatic summaries are the first column and the same summaries edited by humans the second column. Examine these summaries from a scientific and methodical perspective to identify and detail the differences between them. Focus on aspects such as language use, clarity, coherence, structure, and any stylistic variations. Analyze how human editing influences these aspects compared to the automatic generation process. Also include a count of which specific words or phrases are most often deleted or inserted. Please include as much details as possible. Make sure to include three literal quotes from the summaries per observation.
