# Supplementary material for: Impact of a Digital Scribe System on Clinical Documentation Time and Quality: Usability Study
Source: JMIR AI. 2024 Sep 23;3:e60020. doi: 10.2196/60020 (PMC11459111; doi:10.2196/60020)
Supplement: Multimedia Appendix 3 [file ai_v3i1e60020_app3.docx]

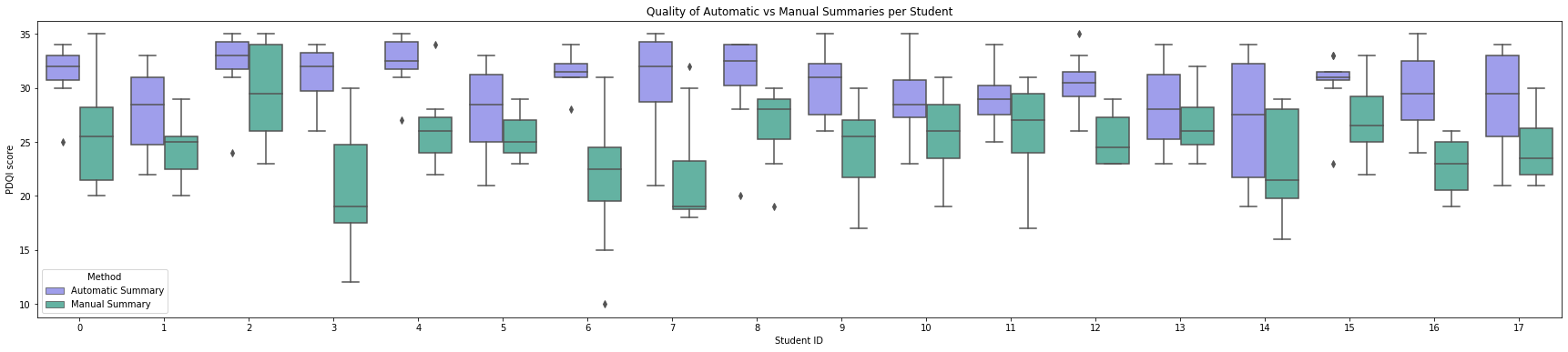


**Figure 1: differences in PDQI score between automatic and manual summaries per student.**


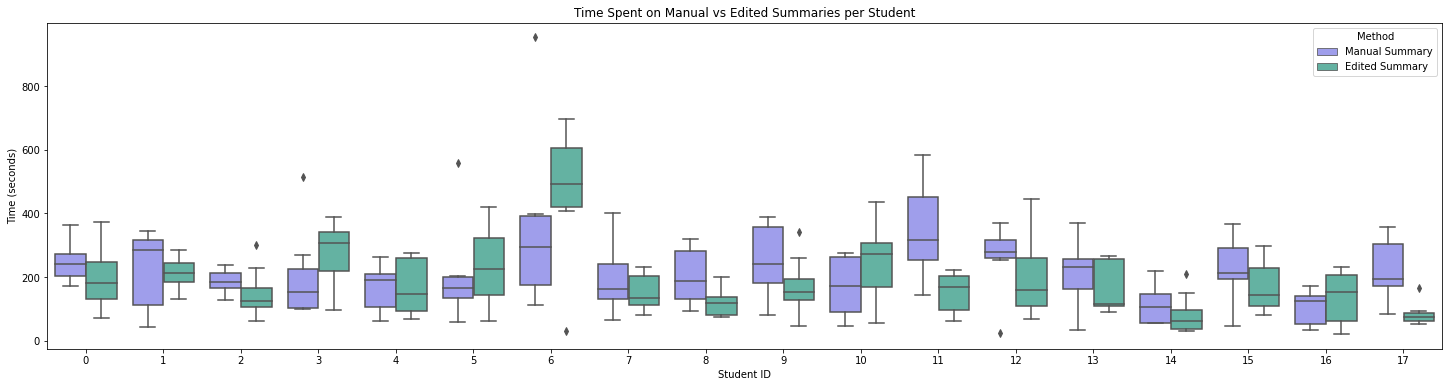


**Figure 2: differences in time spent on manual and edited summaries per student.**
